# Cor‐Knot automated fastener in distal anastomosis of total aortic arch replacement: A case report

**DOI:** 10.1002/ccr3.4555

**Published:** 2021-07-21

**Authors:** Omar M. Sharaf, Tomas D. Martin, Eric I. Jeng

**Affiliations:** ^1^ College of Medicine University of Florida Gainesville FL USA; ^2^ Division of Cardiovascular Surgery Department of Surgery University of Florida Health Gainesville FL USA

**Keywords:** aortic dissection, Cor‐Knot automated fastener, Marfan syndrome, surgical technique

## Abstract

Cor‐Knot fastener use in sternotomy‐based aortic arch procedures has not been reported. We present Cor‐Knot fastener use over a Hegar dilator in an anatomically challenging total aortic arch replacement with no short‐term and/or long‐term complications.

## Graphical abstract

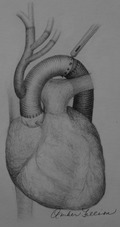

## 1 INTRODUCTION

In patients with connective tissue disorders and vascular manifestations, it is not uncommon to replace the entire aorta in a series of operations. We report the case of a kyphotic gentleman with chronic obstructive pulmonary disease and Marfan syndrome whose history was significant for an emergent thoracoabdominal aortic replacement secondary contained rupture, representing acutely with new acute on chronic chest pain and an acute DeBakey type I aortic dissection. In this anatomically challenging scenario, visualizing the descending thoracic aorta for the distal aortic anastomosis was difficult. We herein present one of the first reports of Cor‐Knot automated fastener utilization in open aortic arch surgery. The patient tolerated the procedure well and was discharged without short‐term, intermediate, and/or long‐term complications.

Acute DeBakey type I aortic dissections frequently present symptomatically at the time of dissection onset and have been associated with risks of aortic rupture, acute severe aortic valve regurgitation, and/or cardiac tamponade.^1^, ^2^ Classically, the treatment for an acute DeBakey type I aortic dissection is with an open surgical repair with an ascending aortic replacement, hemiarch replacement, zone 1 arch, zone 2 arch, and/or total arch replacement. The various approaches for this operation^3^ are balanced with the severity of presentation, immediate mortality risk, facility resources, and surgeon preference. Herein, we present one of first reports of Cor‐Knot automated fastener utilization in open aortic arch surgery.

## 2 CASE PRESENTATION

**FIGURE 1:**
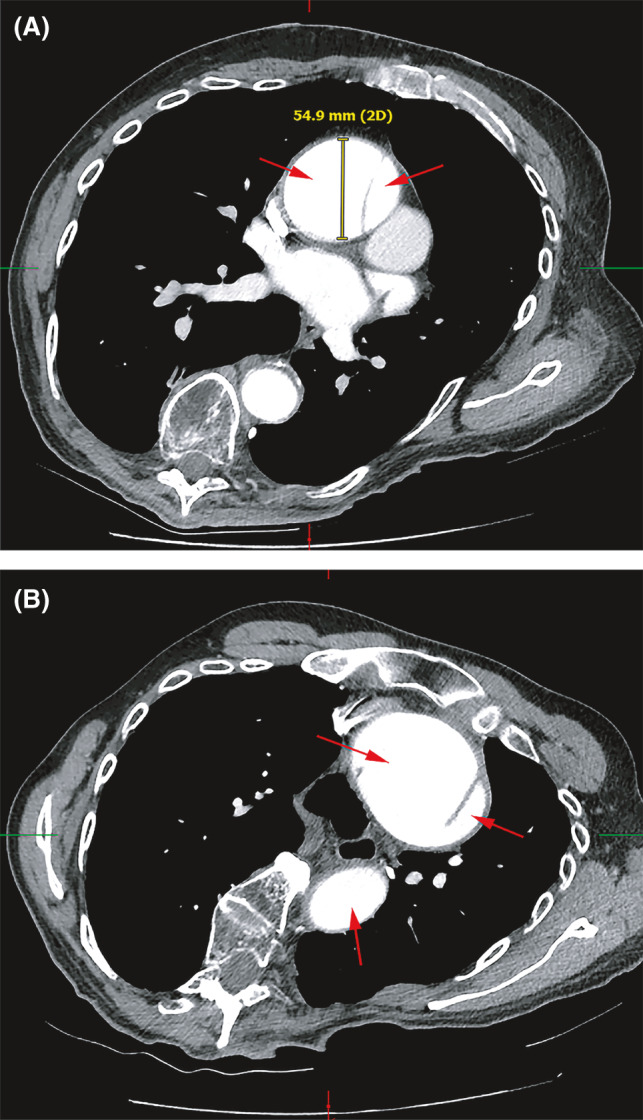
Chest CTA with contrast showing preoperative acute DeBakey type I dissection. (A) Ascending aortic dissection with true and false lumens visualized. Proximal ascending aorta with aneurysmal dilation at 54.9 mm. (B) Descending thoracic aortic dissection with true and false lumens identified. Previous thoracic aorta synthetic dacron graft visualized and intact

A 37‐year‐old gentleman with a past medical history significant for chronic obstructive pulmonary disease (COPD), Marfan syndrome, and a previous open repair for a ruptured Crawford type I thoracoabdominal aortic aneurysm presented to a nearby emergency department with acute on chronic chest pain, altered mental status, and somnolence following cocaine use. He reported that for months, he had syncopal episodes and dysphagia, with chronic chest and back pain, as well as shortness of breath. A computed tomographic angiography (CTA) demonstrated a DeBakey type I aortic dissection, with intact prior descending aortic replacement (Figure 1). On arrival to our institution, he was in sinus rhythm and on anti‐impulse therapy. He was taken emergently for open aortic dissection repair. Through a median sternotomy, we cannulated the aorta centrally with epiaortic ultrasound and modified seldinger technique. Bicaval venous cannulae, retrograde cardioplegia cannulae, and a left ventricular vent through the right superior pulmonary vein were placed. Cardiopulmonary bypass was then initiated with systemic cooling toward 18℃ for deep hypothermic circulatory arrest. The innominate artery was visualized, clamped cephalad, and transected at the level of the aorta for end‐to‐end anastomosis. Antegrade perfusion through the innominate artery was maintained as we completed the left common carotid and left subclavian artery running anastomoses. Our distal aortic anastomosis was completed in Zone 3 of the aortic arch with a portion of the anastomosis to the dacron graft from the prior aortic repair.

**FIGURE 2:**
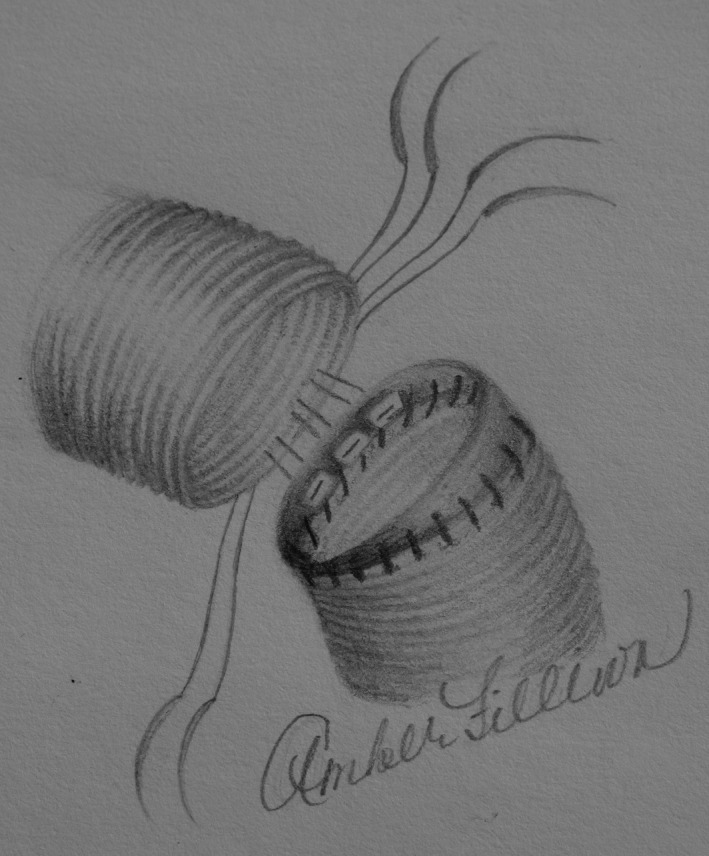
Ethibond valve sutures placed circumferentially in the distal aortic anastomosis before Cor‐Knot fastening

**FIGURE 3:**
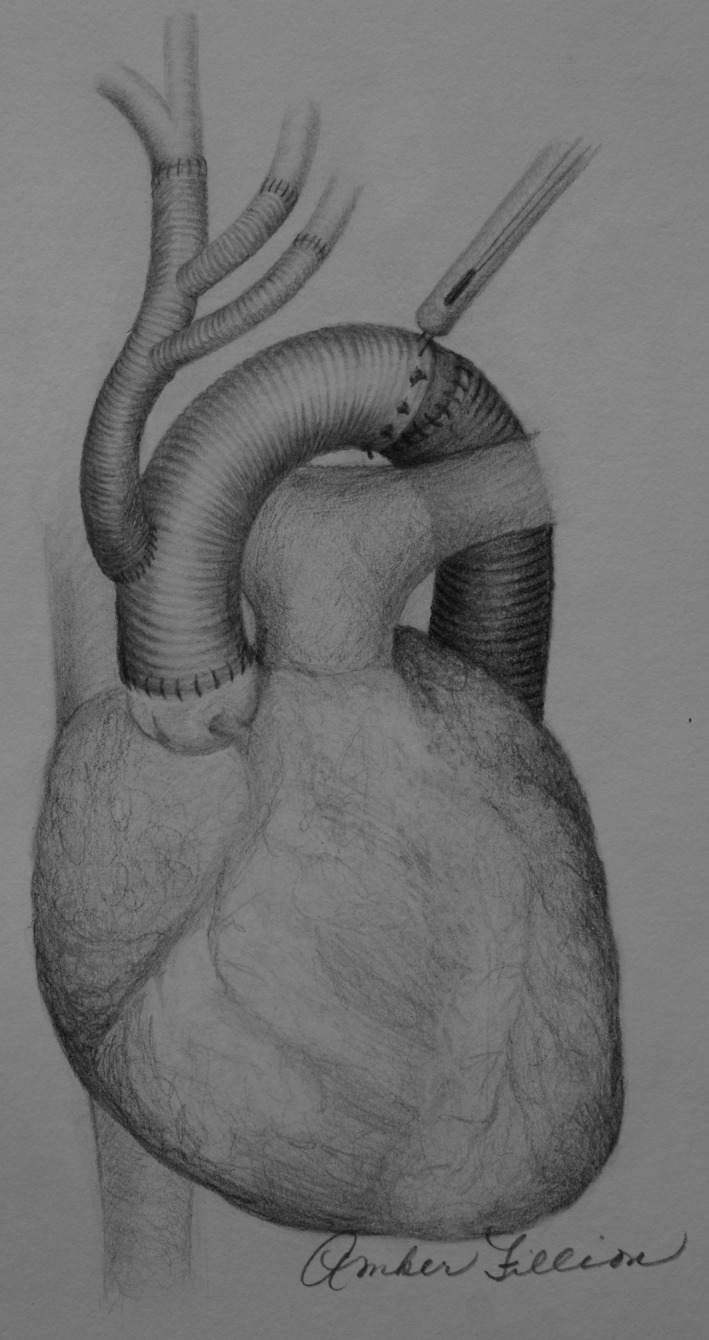
Total aortic arch replacement with trifurcated graft technique and Cor‐Knot automated fastener use in distal aortic anastomosis

In scenarios of normal anatomy, visualizing Zone 3 is frequently difficult via a median sternotomy. The kyphosis and extensive COPD in this patient made an already technically arduous situation virtually impossible with a conventional running aortic anastomotic approach. We placed interrupted 2–0 Ethibond pledgeted valve sutures circumferentially (Figure 2). Once we were satisfied with our suture placement, we completed the anastomosis with the Cor‐Knot automated fastener (Figure 3) over a 22 mm Hegar dilator to prevent aortic coarctation.

Body perfusion was resumed after 75 min, systemic rewarming was started, and the ascending aorta was transected at the sinotubular junction for proximal anastomosis as there was no root and/or valvular pathology. We subsequently completed the debranching anastomosis to the proximal aorta followed by atrial septal defect closure through the right atrium. Deairing was done through the left ventricular vent, the cross‐clamp was removed, rewarming was continued, the patient was decannulated from cardiopulmonary bypass, and the chest was closed. Cross‐clamp time was 197 min, and bypass time was 230 min.

**FIGURE 4:**
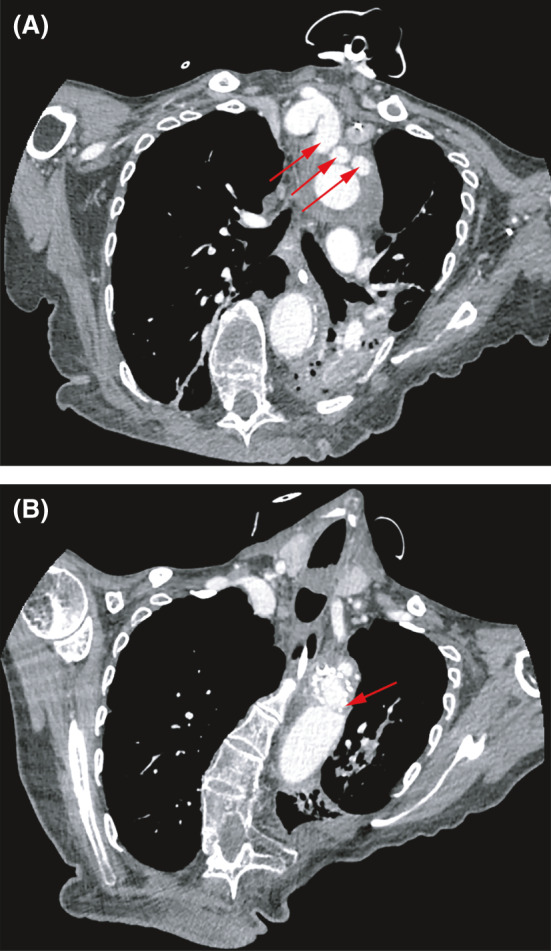
Chest CTA with contrast showing postoperative total aortic arch replacement with Cor‐Knot automated fastening. A) Trifurcated graft anatomy displayed. B) Distal aortic anastomosis with Cor‐Knot automated fastening

Postoperatively, his course was complicated by prolonged intubation, tracheostomy, and bacterial pneumonia. His intensive care unit and total length of stay were 41 and 46 days, respectively. At one‐month follow‐up, he was mildly hypertensive at 135/80 mm Hg with a heart rate of 99 beats per minute. Chest CTA at that time shows trifurcated debranching with Cor‐Knot automated fastening of the distal aortic anastomosis (Figure 4). He had been decannulated from his tracheostomy and had progressively returned to his regular activities. He was scheduled for repeat CTA and follow‐up to be done in one year.

## 3 DISCUSSION

Cor‐Knot fastener is widely used in minimally invasive cardiac valve surgery, but its use in aortic arch surgery has not been reported. In a systematic review of Cor‐Knot use in cardiac valve surgery, Jenkin et al. conclude that significant evidence exists to show that Cor‐Knot use provides intraoperative advantages such as reduced intraoperative times and increased knot strength and consistency.^4^ However, data are lacking as to whether these benefits translate to improved postoperative outcomes.^5^ Cor‐Knot use in sternotomy‐based cardiac valve surgery was evaluated in a randomized clinical trial and was determined to have no significant impact on CPB or cross‐clamp times but with an added financial cost.^6^ In another prospective observational study, the safety of Cor‐Knot automated fastening in patients undergoing aortic valve replacement was comparable to that of patients with manually tied knots as demonstrated by similar 30‐day mortality, stroke and transient ischemic attack rates, pacemaker implantation rates, and rate of aortic regurgitation.^7^ These studies suggest Cor‐Knot is efficacious in minimally invasive cardiac valve procedures where there is limited access and exposure, is safe particularly in aortic valve replacement procedures, but may not provide clinical benefit in sternotomy‐based valve surgery. Cor‐Knot automated fastener use in open aortic arch surgery with limited visualization has not been studied or previously reported.

In this report, a patient with a personal history of connective tissue disease and a prior open descending aortic repair for rupture presented with the surgical emergency for ascending aortic pathology. We elected to perform an aortic dissection repair, total arch debranching, and zone 3 aortic anastomosis. Given limited exposure from a sternotomy approach, coupled patient‐specific characteristics including severe kyphosis and COPD, we performed a zone 3 aortic replacement utilizing interrupted pledgeted sutures and Cor‐Knot fastener. Patients with Marfan syndrome have arterial fragility,^8^ and so utilizing the Cor‐Knot automated fastener has an inherent risk of tear and vascular injury upon deployment. To decrease this risk, our technique utilizes felt plegetts circumferentially to evenly disperse tension. We have employed this technique in a series of subsequent cases with excellent success. Potential pitfalls for this technique are iatrogenic coarctation, which can be combatted by performing the anastomosis over an appropriately sized Hegar dilator. While Cor‐Knot utilization in minimally invasive cardiac surgery is well described, to our knowledge this is the first report describing its employment in open aortic surgery. We believe that many aspects of surgery traverse the intersectional boundaries of science and art, and thus, there is no standard indication for Cor‐Knot usage in sternotomy‐based aortic arch surgery. We utilize and believe that the traditional running prolene and hand tie technique should be the standard, however, show in this report that when presented with unique anatomical situations, the Cor‐Knot can be utilized successfully.

## CONFLICT OF INTEREST

None.

## AUTHOR CONTRIBUTIONS

Omar Sharaf and Tomas Martin: drafted and approved the article. Eric Jeng: critically revised and approved the article.

## ETHICAL APPROVAL

A divisional Institutional Review Board approval for cardiovascular surgery is in place for publication, and informed consent was obtained from the patient.

## Data Availability

The data included herein are available upon reasonable request.
